# Assessment of Ozone Sensitivity in Three Wheat Cultivars Using Ethylenediurea

**DOI:** 10.3390/plants8040080

**Published:** 2019-03-29

**Authors:** Adeeb Fatima, Aditya Abha Singh, Arideep Mukherjee, Tsetan Dolker, Madhoolika Agrawal, Shashi Bhushan Agrawal

**Affiliations:** 1 Laboratory of Air Pollution and Global Climate Change, Department of Botany, Institute of Science, Banaras Hindu University, Varanasi 221005, India; adeebfatima@yahoo.in (A.F.); abha2512.singh@gmail.com (A.A.S.); arideep.mukherjee04@bhu.ac.in (A.M.); tsetan.dolker3@bhu.ac.in (T.D.); madhoo@bhu.ac.in (M.A.); 2 Department of Plant Molecular Biology, University of Delhi, South Campus, Delhi 110021, India

**Keywords:** antioxidants, biomass, ethylenediurea, ozone sensitivity, yield

## Abstract

Three wheat (*Triticum aestivum* L.) cultivars [HD 2987 (ozone (O_3_) sensitive), PBW 502 (intermediately sensitive) and Kharchiya 65 (O_3_ tolerant)] with known sensitivity to O_3_ were re-evaluated using ethylenediurea (EDU; 400 ppm) to ascertain the use of EDU in determiningO_3_ sensitivity under highly O_3_-polluted tropical environments. EDU treatment helped in improving the growth, biomass, photosynthetic pigments and the antioxidative defense system of all the wheat cultivars. Under EDU treatment, PBW 502 retained more biomass, while HD 2987 showed better performance and ultimately the greatest increment in yield. Cultivar Kharchiya 65 also showed a positive response to EDU as manifested with an increase in pigment contents, total biomass and enzymatic antioxidants; however, this increment was comparatively lower compared to the other two cultivars. The results indicated that EDU did not have many physiological effects on cultivars but helped in counteracting O_3_ primarily by scavenging reactive oxygen species and enhancing the antioxidative defense system where superoxide dismutase emerged as the major responsive biochemical parameter against ambient O_3_. The observed results clearly indicated that differential O_3_ sensitivity in three wheat cultivars established by the previous study is in accordance with the present study using EDU as a sensitivity tool, which is an easy and efficient technology in comparison to chamber and Free-Air Carbon dioxide Enrichment (FACE) experiments although its mechanistic understanding needs to be further validated.

## 1. Introduction

Tropospheric ozone (O_3_) is a spatially and temporally dynamic air pollutant as well as a powerful greenhouse gas [1]. This increasing air pollutant of the lower atmosphere has negatively influenced food security, thereby causing tremendous loss to the growth and productivity of various crops. FAO (2009) predicted that, by 2050, the global population will reach 9.1 billion, with a consequent demand of at least a 70% increase in agricultural production and O_3_ will particularly cause much hindrance in achieving this target [2]. The concentration of O_3_ has increased more than two-fold globally in recent times compared to the pre-industrial era [3]. A distinct correlation also exists between the peak O_3_ concentration and major crop growing season, resulting in high yield losses [4]. Global yield losses due to O_3_ in major crops such as wheat, rice, maize and soybean were 4–15%, 3–4 %, 2–5% and 5–15%, respectively [4,5]; however, for the year 2000, it was found that 40% of these losses were from India and China [4]. As per the projection of a new study, a 40% increase in O_3_ concentration is expected in the South Asian region by the year 2050 [6]. Based on mean O_3_ 7h (M7) and accumulated O_3_ over a threshold of 40 ppb (AOT 40) metrics, Lal et al. (2017) estimated an annual total yield loss of 4–14.2 million tonnes (4.2–15%) of wheat production in India during 2011–2014 [7]. However, in the absence of stricter air pollution regulation, India will have to face more crop yield losses in the future due to O_3_ pollution.

Different techniques and chemical tools have been used to assess the level of economic losses from O_3_ exposure, of which ethylenediurea (EDU, N-[2-(2-oxo-1-imidazolidinyl) ethyl]-N′-phenylurea) has widely emerged as a clear indicator in the assessment of EDU-induced protection in plants against O_3_. It was first used by Carnahan et al. (1978), and showed a positive effect on plant productivity against ambient O_3_ [8]. EDU-dependent O_3_ tolerance occurs by modifying plant processes at the cellular level, ultimately protecting plant yield from O_3_ damage [9]. Feng et al. (2018) found EDU to be effective in increasing yield which decreased by 20.3% in non-EDU-treated wheat cultivars, and it has also been used to screen for O_3_-sensitive/tolerant cultivars [10]. EDU treatment (500 ppm) to Sesame increased stomatal conductance (g_s_) and the net photosynthesis rate (P_s_) by 52 and 61%, respectively, compared to plants without EDU treatment [11], while Singh et al. (2010) noticed higher ascorbic acid content in mung bean plants with EDU application, and peroxidase (POX) and superoxide dismutase (SOD) activities were significantly declined [12]. A recent study by Pandey et al. (2019) demonstrated that EDU can be widely used in large scale screening for O_3_ tolerance in different wheat cultivars under different environments [13]. EDU thus effectively protects and also helps in estimating the extent of O_3_ damage to crop plants. To date, O_3_ sensitivity is identified in different cultivars based on chamber and Free-Air Carbon dioxide Enrichment (FACE) experiments which are costly and require sufficient instrumentation, while sensitivity assessment using EDU under ambient conditions is rather effective and can be directly used for screening in developing countries.

Wheat is one of the most important staple crops in the world after maize [14] and its sensitivity to O_3_ is known to be cultivar specific [9,15]. Thus, this present study was conducted on three tropical wheat cultivars to assess the severity of O_3_ damage using EDU as a chemical tool. We hypothesized that EDU will help in validating the cultivar-specific O_3_ sensitivity of wheat and also change either the physiological parameters or the antioxidative defense system, contributing to variations in yield under ambient O_3_ conditions. Therefore, we used EDU as a tool with the following objectives: (1) to confirm the relative sensitivity of selected wheat cultivars already categorized to be sensitive, intermediately sensitive and tolerant against O_3_, (2) to assess whether these cultivars differ in their physiological or biochemical responses to O_3_, and (3) to find the most influential parameters of O_3_ sensitivity in wheat cultivars. This knowledge will help in validating the use of EDU in the screening of cultivars for O_3_ sensitivity for cultivation in a particular area and identification of some important parameters present in tolerant cultivar for breeding purposes.

## 2. Results

The mean monthly ambient O_3_ concentration of 56.7 ppb (maximum) was observed in March and a minimum of 46.8 ppb was observed in February, while the seasonal mean O_3_ concentration was 49.6 ppb. Likewise, the maximum AOT 40 value of 2939.4 ppb h was observed in the month of March and the cumulative AOT 40 value observed for the ambient O_3_ environment was 9168.21 ppb h during the experimental period.

Growth parameters (plant height, total biomass and number of tillers) were positively influenced by EDU treatment in all the tested cultivars (Table 1 and Table 2). At 60 days after germination (DAG), the plant height increased maximally by 14.4% in HD 2987, while it increased least in Kharchiya 65, by 6.2%.The number of tillers was maximally increased in PBW 502 by 57.1% and it increased least in Kharchiya 65, by 31.8%, while the total biomass increased by 34.3% in PBW 502 followed by 22.3% in HD 2987 and least in Kharchiya 65 by 12.2%.A split-plot ANOVA also found a significant effect of EDU treatment and cultivars for all the growth parameters (Table 1). Among the growth parameters, the total biomass showed the maximum influence of EDU treatment while the number of senesced leaves was least affected. The interactive effect of EDU treatment and cultivars were only significant (*p* < 0.05) for biomass and the number of senesced leaves.

Under EDU treatment, P_s_ increased maximally by 34.4% in HD 2987 (*p* < 0.05) and least in Kharchiya 65 by 9.7%, while g_s_ increased maximally by 26.5% in Kharchiya 65 and minimally in HD 2987 by 17.3% (Table 2). Chlorophyll fluorescence, i.e., Fv/Fm, was maximally increased in HD 2987 by 12% and minimally by 1.4% in Kharchiya 65, although the response was non-significant under EDU treatment. Among the physiological parameters, the maximum significant (*p* < 0.05) effect of EDU treatment was observed in Ps while g_s_ was least affected; however, the effect of the cultivar was non-significant (*p* = 0.078) in the case of Ps (Table 1). For all the physiological parameters, the effect of the interaction between EDU treatment and cultivars were non-significant (*p* > 0.05).

**Figure 2:**
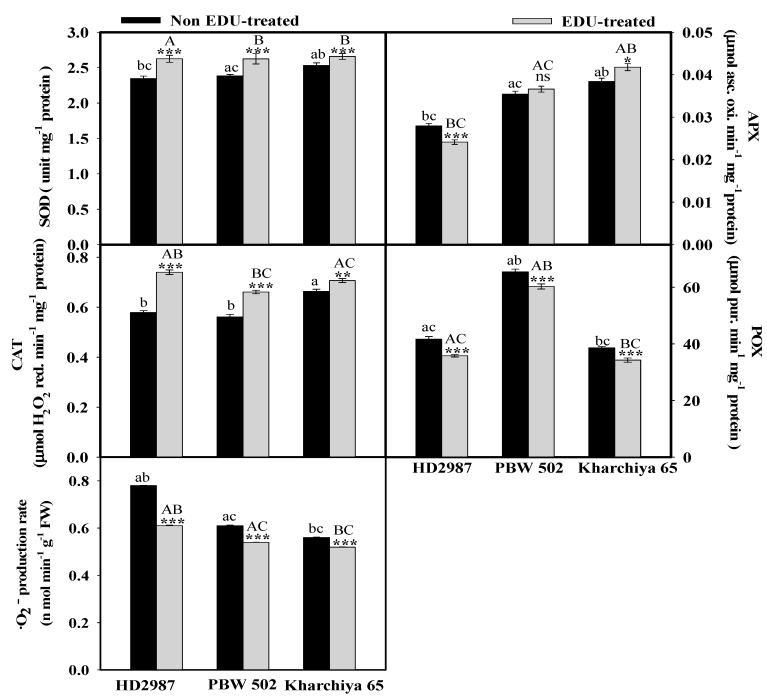
Effect of EDU on antioxidative enzymes such as superoxide dismutase (SOD), ascorbate peroxidase (APX), catalase (CAT), peroxidase (POX) and the superoxide radical production rate (SOR) in wheat cultivars. Values are mean ± SE; n = 9. Symbols above the bars represent significant variability between EDU- and non-EDU-treated plants. The one, two and three asterisks indicate the significance levels at * *p* ≤ 0.05, ** *p* ≤ 0.01, and *** *p* ≤ 0.001, and ns is non-significant. Significant variations between cultivars under EDU and non-EDU treatment are respectively represented by different capital and small letters (*p* < 0.05).

In the EDU-treated cultivars, the enzymatic antioxidants displayed a variable trend. The results of split-plot ANOVA also revealed a significant effect (*p* < 0.001) of the EDU treatment, cultivars and their interaction on all the selected enzymatic antioxidants except APX (Table 1). SOD was found to be the most affected parameter by EDU treatment (Table 1). The maximum increase in SOD activity was in HD 2987 (11.9%), while the least induction was in Kharchiya 65 being 5% in EDU-treated plants (Figure 2). The superoxide radical production rate (SOR) was significantly and maximally reduced by 21% in HD 2987, followed by 11.6 and 6% in cultivars PBW 502 and Kharchiya 65, respectively. Catalase (CAT) activity also showed a trend of increase after EDU application, being highest in HD 2987 (27%) and lowest in Kharchiya 65 (6.5%). POX activity reduced maximally in HD 2987, by 14.1%, while it decreased by 7.7% and 11.1% in PBW 502 and Kharchiya 65, respectively. APX activity declined after EDU supplementation in HD 2987 by 13.5%. However, an induction in the APX activity was in Kharchiya 65 (8.4%) and there was a non-significant increase of 0.4% in PBW 502 upon EDU treatment compared to the non-EDU-treated ones (Figure 2).

**Figure 3:**
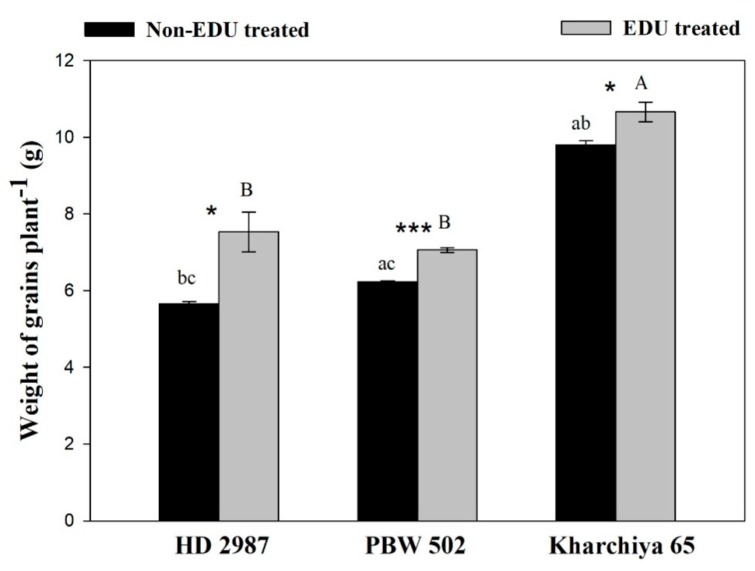
Yield (weight of grains per plant) of three wheat cultivars as affected under EDU treatment. Values are mean ± SE; n = 9. Symbols above the bars represent significant variability between EDU- and non-EDU-treated plants. The one, two and three asterisks indicate the significance levels at * *p* ≤ 0.05, ** *p* ≤ 0.01, and *** *p* ≤ 0.001, and ns is non-significant. Significant variations between cultivars under EDU and non-EDU treatment are respectively represented by different capital and small letters (*p* < 0.05).

EDU application resulted in an improvement in the yield (weight of grains plant^−1^) of HD 2987 (32.9%), PBW 502 (13.3%) and Kharchiya 65 (8.8%). Therefore, EDU application confirmed its suitability for identifying the cultivar susceptibility against O_3_ stress considering the economic yield (Figure 3).

**Figure 4:**
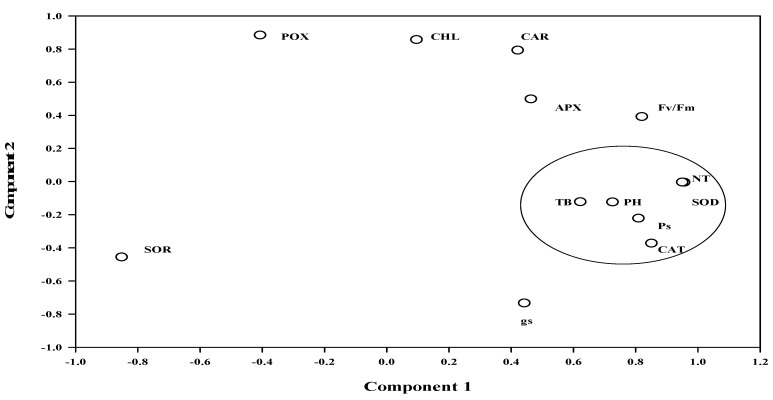
Principal component analysis results showing an association of the parameters on component axes. Here, PH, NT, TB, Ps, g_s_, Fv/Fm, CHL, CAR, SOD, POX, CAT and APX are the plant height, number of tillers, total biomass, photosynthetic rate, stomatal conductance, photosynthetic efficiency, chlorophyll, carotenoids, superoxide dismutase, peroxidase, catalase and ascorbate peroxidase, respectively.

Based on the PCA results (Figure 4), the total variance explained by three components was 89.7%, while it was 48.2%, 26.9% and 14.5% by components 1, 2 and 3, respectively. Parameters such as SOD, CAT, plant height, number of tillers, total biomass and P_s_ were closely related irrespective of other parameters showing a high association between them. In component 1, SOD showed the highest loading of 0.937 followed by a number of tillers (0.930).

## 3. Discussion

EDU has been verified as a simple and effective chemical tool to evaluate and assess O_3_ phytotoxicity under ambient conditions [16,17]. During the growing season of wheat, the high mean ambient O_3_ and AOT 40 values in our experiment were well correlated to the concentrations reported by other studies from India [9,18], having a significant effect on the growth and productivity of various crops.

In our experiment, EDU-treated wheat cultivars showed significant enhancements in plant height and number of tillers, indicating O_3_ stress-induced suppression in growth characteristics. Moreover, plant growth characteristics were highly improved under EDU treatment in both the sensitive and intermediately sensitive cultivars. Tiwari et al. (2005) also found improved morphological characteristics (plant height and number of tillers) in EDU-treated wheat cultivars [19] while under 50 ppb O_3_; EDU application of 400 ppm caused a significant rise in height of mung bean plants [20]. Among the morphological traits, only the total biomass and number of senesced leaves showed significant variability due to the interactive effect of both treatment and cultivar. Such results indicate that both of these parameters depend upon EDU treatment with different magnitudes, suggesting that the intrinsic defense mechanism and resource utilization strategies are differentially altered by EDU in different cultivars in the presence of EDU.

We also found increments in the photosynthetic pigments under EDU treatment compared to non-EDU-treated plants. Besides, the sensitive cultivar showed significantly higher chlorophyll content in comparison to two other cultivars under EDU treatment. As the sensitive cultivars are more shielded to the negative influence of O_3_ compared to other cultivars, O_3_ stress might have induced more pigment synthesis instead of an induction in defense response. The results observed in mustard cultivars, Kranti and Peelasona, also showed increased chlorophyll content upon EDU treatment owing to the reduced destruction of chlorophyll [21]. The positive effect of EDU on photosynthetic pigments is well documented [12,22]. Pre-treatment with EDU has been conferred to protect against the O_3_-induced loss of chlorophyll [23]. Another pigment (i.e., carotenoids) imparts protection against photo-oxidative damage by the effective quenching of free radicals and, thus, helps in maintaining higher contents of chlorophyll. Therefore, increased carotenoids content upon EDU treatment would also have helped the plants in maintaining higher chlorophyll content for a long time. Similarly, EDU-treated soybean plants showed increased total chlorophyll and carotenoids content by 13.6% and 12.1%, respectively, than non-EDU-treated plants [22]. In the present experiment, plants of tolerant cultivar Kharchiya 65 showed an increase in carotenoids compared to the other two cultivars. Another study also reported greater carotenoids content in Pinto bean, depicting a direct effect of EDU (500 mg L^−1^) treatment on them [24]. All three wheat cultivars displayed a delay in senescence under EDU treatment and it was keenly observed that one of the effective means by which EDU conferred protection to cultivar HD 2987 was by maintaining higher chlorophyll content under ambient O_3_ conditions. EDU has been known to have a cytokinin-like mode of action and, thus, helped to retain chlorophyll by minimizing oxidative stress and delaying the process of senescence [25]. Additionally, an increase in carotenoids content also supports the anti-senescent property of EDU as it also helps in preventing chlorophyll damage from photo-oxidation during stress conditions.

Ozone causes a reduction in C assimilation due to decreased Rubisco activity or impaired stomatal function, leading to a decline in C availability in leaves [26]. Under EDU treatment, the sensitive cultivar showed a significant rise in P_s_, reflecting that EDU effectively maintains a higher rate of P_s_. However, in the case of other two cultivars, there were no significant effects on P_s_ EDU application. Higher P_s_ in the sensitive cultivar is also directly correlated with higher pigment and carotenoids content under EDU treatment compared to other cultivars. Higher Ps and total chlorophyll content might be the reason for the increased total biomass of the sensitive cultivar in our study which was also confirmed in previous studies [27]. Tiwari et al. (2005) found a significant positive influence on total biomass as well as on shoot, leaf and root weights of wheat cultivars [19] whereas another study showed a 24% increment in the total biomass of mung bean plants after EDU supplementation [20]. Similar findings were also reported by Pandey et al. (2014) in cultivars of *Brassica campestris* [21] and by Singh et al. (2018) in maize cultivars [28]. The present study showed that EDU supplementation helped the wheat plants to maintain more biomass under ambient O_3_ conditions. It is a matter of great interest as to how, without a significant increase in photosynthesis, EDU-treated plants grew better and accumulated more biomass. This could be well satisfied with the explanation that reduced resource utilization for the antioxidative defense system under EDU treatment supported more growth and the biomass characteristics of wheat plants. Most results from earlier works suggest a non-significant effect of EDU on photosynthesis [23,29]. A meta-analytical study revealed that EDU-mediated protection against O_3_ was biochemical rather than biophysical [23]. Due to the complex nature of O_3_ action on stomata, there is no specific EDU effect on stomatal conductance [30]. Although in the present study, g_s_ was partially influenced by EDU treatment only in the intermediately sensitive cultivar while the effect was non-significant for the sensitive as well as the tolerant cultivars due to EDU application, which again highlights the fact that EDU might influence plants by its protective mechanism and not by enhancing or altering different physiological processes or the entry of O_3_ through stomata. Rai et al. (2015) recorded significant increases in the Fm and Fv values in EDU-treated plants compared to non-EDU-treated plants, whereas the Fo and Fv/Fm ratio did not vary significantly between the two treatments [22]. We also observed a similar finding where Fv/Fm did not show a significant response across all test cultivars under EDU treatment, although the overall effect was significant and that might be due to different reasons such as the antioxidative status of different cultivars, photosynthetic repair mechanisms and accessory pigment contents. The biophysical and physiological parameters tested in the present study were not influenced by EDU treatment in all of the cultivars, suggesting that a single physiological process is not the direct target of EDU in enhancing stress tolerance against O_3_. Instead, the EDU response appears to involve a complex interaction of processes that modulate plant physiological efficiency.

It has been hypothesized that EDU results in less C investment in the antioxidative defense mechanism for the repair of O_3_-induced damage and a major proportion of C can be utilized for growth and development [31]. The responses of antioxidant enzymes were in accordance with the results observed in the literature for other species treated by EDU [17,28,32]. Rai et al. (2015) found significant increases of 11.1% and 18.5% in activities of SOD and APX in EDU-treated than non-EDU-treated soybean plants [22]. In our experiment, both SOD and CAT were highly responsive in the sensitive cultivar. Likewise, SOD and CAT activities were also induced in the sensitive clover cultivar Wardan under EDU treatment [33]. Lower accumulation of ^·^O_2_^−^ radical in all the cultivars is directly related to the differential activities of different antioxidative enzymes upon EDU treatment. It was demonstrated that EDU halted reactive oxygen species (ROS) production in *Phaseolus vulgaris* L. plants within 24 h of O_3_ treatment, resulting in decreased H_2_O_2_ production [34]. Possible modes of EDU action are the direct scavenging of ROS generated by O_3_ [35] or up-regulation of the antioxidative defense system in plants [23,31]. Supporting this, the present study showed a higher stimulation of antioxidative enzyme activityupon EDU treatment, particularly, in the activity of SOD, which is normally associated with O_3_ tolerance. While both SOD and CAT activities were found to be the most affected parameters across 18 rice cultivars when assessed with EDU against a high O_3_ concentration [17]. Besides, the other relevant enzyme POX decreased in all the assessed wheat cultivars under EDU treatment as also observed by Tiwari and Agrawal (2010) [27]. They also discarded EDU as a potent antioxidant but emphasized its role in maintaining higher levels of antioxidants under O_3_ stress with better physiological performance [27]. It is a well-known fact that, under EDU treatment, the sensitive plants showed significant responses to O_3_, while tolerant plants showed only limited responses [32,36]. The ozone sensitivity of different cultivar types such as for wheat [32,37] and rice [38] cultivars are widely known. We also found an interactive effect of both treatment and cultivar for all the studied enzymes, which again highlights the fact that EDU alteration is directly influenced by the O_3_ sensitivity of different cultivars [10,39].

The mechanism of EDU action differed in all the cultivars as P_s_, chlorophyll, SOR, SOD and CAT were maximally affected in HD 2987, while it maximally enhanced biomass in PBW 502; furthermore, antioxidative enzymes were induced upto certain limits. Apart from these two cultivars, Kharchiya 65 was maximally influenced with higher carotenoids content under EDU treatment with the lowest induction of antioxidative enzymes.

The ameliorative role of EDU was manifested on yield under ambient O_3_ conditions, which showed a comparatively higher yield with respect to control. EDU supplementation helped the wheat plants in translocating more photosynthates to their reproductive parts and, therefore, played a significant role to minimize yield losses. Similar to this, a study performed in China also showed an increase in the yield characteristics of wheat after EDU treatment [40]. Feng et al. (2018) also reported >25% yield reduction in the sensitive cultivars and <10% for the tolerant ones [10]. The three wheat cultivars differed in their resource allocation strategy. The higher gain of yield may simply be explained by better defense due to the efficient induction of the antioxidant system as observed in HD 2987 (with comparative lower increase in biomass), while the lower gain of yield may be related due to a trade-off between biomass accumulation and the translocation of photosynthates to reproductive parts as in PBW 502, resulting in greater biomass at the cost of grain weight due to EDU treatment. Also greater biomass in PBW 502 can be attributed to other physiological or biochemical events in the developmental stages of this cultivar. It has also been shown that the sensitive cultivar allocates more of its resources towards defense actions in response to O_3_-affected reduction in biomass [18]. Although Kharchiya 65 showed a positive response under EDU treatment, that was the lowest because the constitutive mechanisms in this cultivar were already much more efficient in tolerating O_3_ stress.

The overall results proved the usefulness of EDU as a tool to monitor cultivar-specific sensitivity to ambient O_3_. A recent study by Pandey et al. (2019) also confirmed the use of EDU as an efficient tool to reveal the adverse impacts of O_3_ eleven wheat cultivars and also helped to classify their relative sensitivity/tolerance to O_3_ [13]. This classification of cultivars will provide useful information for supporting the selection of the best-suited cultivars in areas with different O_3_ concentrations. The sensitivity in different cultivars was mostly due to the differential responses of different physiological and biochemical defense responses. EDU protects the plant by delaying the process of senescence, enhancing growth, biomass and economic yield, although the magnitude of these effects was cultivar specific. The present study confirms our earlier observation of the wheat cultivar sensitivity to O_3_. These results suggest EDU should be more frequently used as a tool for biomonitoring in such types of studies in the absence of sophisticated chamber experiments [30,31]. Our hypothesis was partly confirmed as EDU helped in mitigating the deleterious O_3_ effect through a different mechanism in the differentially sensitive cultivars, although conclusive evidence of how EDU protects plants still needs to be resolved. Unlike experiments using open top chambers (OTCs), EDU experiments can effectively provide estimations of the extent of damage to crop yields against O_3_ and the effects on other parameters without bearing complexities associated with the micro-environment as faced during enclosure techniques [30,31] while providing similar results as also observed in OTC studies. Similar to our previous study [9], the present study also found total plant biomass to be important trait in identifying O_3_ sensitivity in different cultivars, which was also found to indirectly influence the overall yield response. This study helped in validating the cultivar response and O_3_ sensitivity using a cheap method with no set-up requirement.

## 4. Materials and Methods

### 4.1. Experimental Site and Plant

The experiment was executed during the wheat growth period, extending from mid-December 2015 to the end of March 2016, at the experimental field of the botanical garden in the campus of Banaras Hindu University, Varanasi, India, at an altitude of 76 m a.s.l. and located in the Eastern Gangetic plains of North India—with an overall soil texture that includes 45, 28 and 27% sand, silt and clay, respectively, and has a pH ranging from 7.2–7.4. Based on the prior screening experiment, three wheat cultivars (*T. aestivum* cvs) with different levels of O_3_ sensitivity were selected, namely, HD 2987 (sensitive), PBW 502 (intermediately sensitive) and Kharchiya 65 (tolerant) [9]. HD 2987 had a plant height of 86–94 cm, a maturity period of 128–134 days and was drought tolerant. PBW 502 had a plant height of 80–94 cm, a maturity period of 128–139 days and was tolerant to heat and lodging, while Kharchiya 65 had a plant height of 111–130 cm, a maturity period of 132–142 days and was salinity/alkalinity tolerant. The average yield varied from 3.2, 4.6 and 4.2 tonnes/hectare for HD 2987, PBW 502 and Kharchiya 65, respectively.

### 4.2. Raising of Plants and EDU Treatment

**Figure 5:**
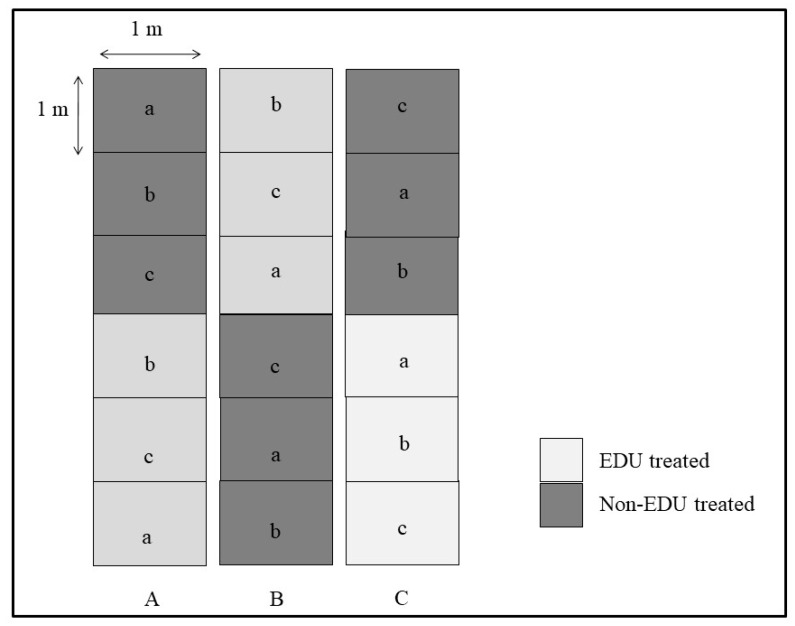
The layout of the whole plot, consisting of three main blocks (A, B and C) with twosub-plots (EDU-treated and non-EDU-treated) and a further threesub-subplots (a, b and c). Here, a, b and c are three different wheat cultivars (HD 2987, PBW 502 and Kharchiya 65).

The experimental plot (split-split plot design) was an open plot consisting of three blocks, namely, A, B and C (separated by 0.2 m from one another). Each block was then further separated into two sub-blocks (EDU- and non-EDU-treated). Each sub-block was further divided into three blocks or sub-subblocks (1 × 1 sq. m dimensions) represented by each of the three cultivars (a, b, c) (Figure 5). The entire plot was prepared using Indian standards of agronomic practices. [41]. During the field preparation, urea, muriate of potash and superphosphate were added (120, 40 and 80 kg ha^−1^, respectively) as the source of N, K and P, respectively. Seeds were manually sown in the first week of December inside each block and, after germination, a total of 36 plants were maintained in each plot. Dr. Lisa Emberson, of Stockholm Environment Institute, University of York, UK, provided EDU as a gift. A freshly prepared EDU solution (400 ppm; prepared using 400 mg EDU in lukewarm distilled water with a final volume adjusted to 1 L) was given as a soil drench to each plant (in EDU treatments) at an interval of every 10 d while the first dose was applied at 10 DAG. This prescribed concentration of EDU most effectively showed positive results on field crops [23] and was, therefore, used in our experiment. In EDU treatments, up to 40 DAG and 100 mL EDU solution were added with subsequent addition of 200 mL until there were 90 DAG, making up a total of nine applications to individual plants. Control plants (no EDU treatment) were provided with a similar amount of water for maintenance of equal water regime.

### 4.3. Meteorological Data

Meteorological data collected during the similar study period showed the average maximum temperature of 33.5 °C in March and the average minimum temperature in January (10.2 °C). Seasonal average relative humidity during the study period was 55.7%, while the average number of bright sunshine hours was 6.5 h with a maximum of 8.7 h in the month of March. All data were collected from the Indian Meteorological Division (IMD) located in the Banaras Hindu University campus.

### 4.4. Ozone Monitoring

The ambient O_3_ concentration at the experimental site was monitored using an automatic O_3_ analyzer (Horiba, APOA-370, Japan) on eight h day^−1^ from 09:00 to 17:00 h. Air samples (0.72 L/min) were drawn in through a Teflon tube (0.35 cm in diameter and 4 cm in height) above the plant’s canopy. For the ambient O_3_ concentration, mean hourly values were calculated followed by the calculation of mean monthly O_3_ concentration. The exposure index for O_3_, i.e., AOT 40, was calculated by the formula given by Mauzerall and Wang (2001) [42].

$$\mathrm{AOT}40=\overset{n}{\underset{i=1}{\sum }}[{\mathrm{Co}}_{3}-40]$$

where, ${\mathrm{Co}}_{3}$ denotes the mean O_3_ values per hour ppb, *i* is the index, and *n* indicates the number of hours where O_3_ values were above 40 ppb.

### 4.5. Growth Parameters and Total Biomass

Plants with intact roots were uprooted at 60 DAG for assessing the growth parameters and total biomass. Five plants per cultivar were taken randomly from sub-subplots of each treatment and were thoroughly washed under tap water to remove all adhered soil particles. Further, the growth parameters such as the height of the plant, number of tillers plant^−1^, number of leaves and senesced leaves plant^−1^ were recorded. Plant parts such as the root, stem and leaves were first separated and then dried in an oven at 80 °C until constant weights were achieved.

### 4.6. Physiological Parameters

Aportable photosynthetic system (LICOR/LI-6400 XT Biosciences, Inc., Lincoln, NE, USA) was utilized to assess P_s_ and g_s_ on fully expanded third leaves from the top at 60 DAG. These measurements were recorded on cloud-free days during 09:00 and 10:30 h and five plants cultivar^−1^from three sub-subplots of each treatment were chosen randomly for measurement. Leaves were illuminated with a photosynthetic photon flux density (PPFD) of 1200 μmol m^−2^ s^−1^ using an internal light source of the leaf chamber. The leaf temperature, flow rate and CO_2_ concentration were 25.0 °C, 300 μmol s^−1^ and 400 ppm, respectively. Chlorophyll, a fluorescence or photosynthetic efficiency (Fv/Fm), was measured for the different treatments using the Portable Plant Efficiency Analyzer (PEA, Hansatech Instument Ltd., UK) on the tagged leaves where gas exchanges were measured. Leaf clips were attached on the adaxial leaf surfaces for adaptation under dark condition (30 min) and then measurement was taken at 3000 μmol m^−2^ s^−1^ irradiance excitation.

### 4.7. Biochemical Parameters

For all biochemical parameters, fully expanded third leaves from the top (n = 3) were taken at 60 DAG from the respective sub-subplots of each treatment. Photosynthetic pigments were estimated by taking 0.1 g fresh leaf samples which were homogenized in 80% acetone and the absorbance was recorded at 480 and 510 nm for carotenoids content and at 645 and 663 nm for chlorophyll a and b. The formula by Maclachlan and Zalik (1963) was used for the calculation of chlorophyll content and the formula given by Duxbury and Yentsch (1956) was used for calculating carotenoids content [43,44]. SOR (O_2_^−^) was estimated following the rate of nitrite formation by reacting with hydroxylamine in the presence of O_2_^−^ [45]. For this, 0.5 g leaf sample was homogenized in 3 mL of 65 mM phosphate buffer (pH–7.8) and the final absorbance of the pale-yellow colored solution was measured at 530 nm. Antioxidative enzymes (SOD, ascorbate peroxidase (APX), catalase (CAT) and POX) activities were assayed and calculated following the methodologies already provided by Singh et al. (2014) [46].

### 4.8. Yield

For estimation of the yield, the weight of the grains per plant was estimated after 135 DAG. Fully mature healthy grains were considered form three different plants of each cultivar from each sub-subplots (n = 9).

### 4.9. Statistical Analyses

Before the statistical analysis data were checked for normal distribution by the Kolmogorov–Smirnov test and the homogeneity of variance was checked by Levene test, the non-normal data were log transformed to meet the assumptions of the analysis of variance (ANOVA).To test the main effect of EDU treatment, cultivars and their interaction on different studied parameters (dependent variable), a mixed-design analysis of variance or split-plot ANOVA were performed. To test the significant variability between EDU- and non-EDU-treated plants for each cultivar univariate, ANOVA was performed followed by Tukey’s post hoc test to assess variations among the three different cultivars. For each of the analyses, each sub-subplot was taken as a statistical unit. To identify the association between different parameters tested, principal component analysis (PCA) was executed utilizing the correlation matrix and varimax rotation method. All the statistical tests were undertaken using SPSS software (SPSS Inc., version 23.0).

## 5. Conclusions

EDU application was found to be a useful tool in the ranking of cultivar-specific sensitivity in the present study. EDU helped wheat plants accumulate more biomass under ambient O_3_ conditions. The most sensitive cultivar, HD 2987, showed better performance under EDU treatment and ultimately led to a maximum increment in yield. EDU had no direct physiological effect but helped to mitigate the deleterious O_3_ effect primarily by scavenging ROS and enhancing the antioxidative defense system, thus delaying senescence and, thereby, reducing chlorophyll loss and enhancing the growth, biomass and yield of wheat cultivars. It was also found that the mechanisms of their relative sensitivity were not similar in different cultivars and SOD emerged as the major responsive biochemical parameter in providing protection against ambient O_3_. Thus, the short-term adaptive strategies may include the cultivation of wheat cultivars that are both O_3_tolerant and high yielding, while the most O_3_-sensitive cultivars should be avoided.

## Figures and Tables

**Figure 1:**
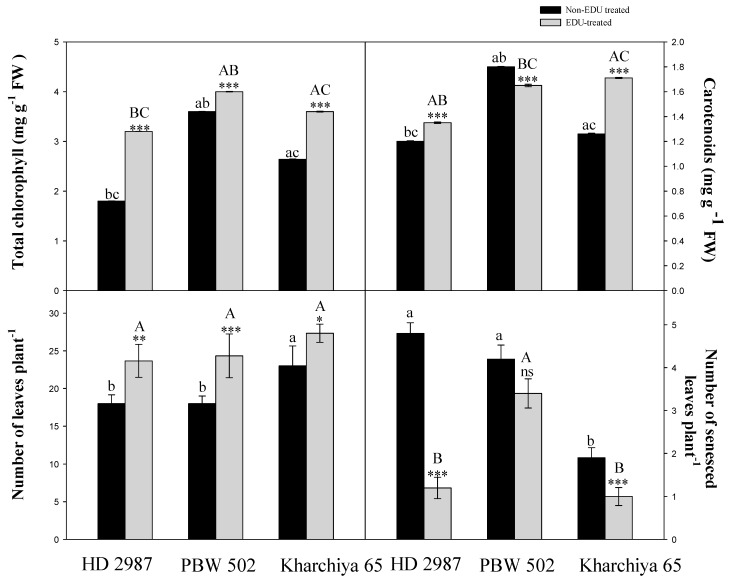
Effect of EDU treatment on total chlorophyll, carotenoids, number of leaves per plant and number of senesced leaves per plant in three cultivars of wheat. Values are mean ± SE; n = 9. Symbols above the bars represent significant variability between EDU- and non-EDU-treated plants. The one, two and three asterisks indicate the significance levels at * *p* ≤ 0.05, ** *p* ≤ 0.01, and *** *p* ≤ 0.001, and ns is non-significant. Significant variations between cultivars under EDU and non-EDU treatment are respectively represented by different capital and small letters (*p* < 0.05).

**Table 1:** Results of the split-plot ANOVA with their respective df (degrees of freedom), *F* (*F*-value) and *p* (significance level) values for each of the studied parameters (n = 9). EDU treatment and cultivar are individual factors while EDU × Cultivar is the interactive factor. Here, P_s_: photosynthesis rate, g_s_: stomatal conductance and Fv/Fm: photosynthetic efficiency, APX: ascorbate peroxidase, POX: peroxidase, CAT: catalase and SOD: superoxide dismutase.

Parameters	EDU Treatment			Cultivar			EDU × Cultivar		
	df	*F*	*p*	df	*F*	*p*	df	*F*	*p*
Total biomass	1	154	<0.001	2	186	<0.001	2	15.34	0.014
Plant height	1	17.39	0.013	2	54.55	<0.001	2	2.76	0.122
No. of tillers per plant	1	60.5	0.001	2	19.92	0.001	2	0.071	0.932
No. of senesced leaves	1	5.56	0.078	2	19.85	0.005	2	10.42	0.018
No. of leaves	1	10.91	0.030	2	2.935	0.111	2	0.131	0.879
Total chlorophyll	1	207	<0.001	2	219	<0.001	2	314	<0.001
Carotenoids	1	119	<0.001	2	256	<0.001	2	335	<0.001
P_s_	1	59.1	0.002	2	4.03	0.073	2	0.76	0.482
g_s_	1	9.04	0.040	2	17.99	0.005	2	0.385	0.690
Fv/Fm	1	40.79	0.003	2	10.34	0.013	2	3.032	0.105
SOD	1	374.1	<0.001	2	300.2	<0.001	2	138.6	<0.001
POX	1	121.3	<0.001	2	227.2	<0.001	2	197.5	<0.001
CAT	1	106.9	<0.001	2	107.1	<0.001	2	66.9	<0.001
APX	1	0.205	0.674	2	145.1	<0.001	2	33.95	0.001
Superoxide radical Production rate	1	87.11	<0.001	2	111.4	<0.001	2	91.95	<0.001
Yield	1	134	<0.001	2	103.9	<0.001	2	2.064	0.219

**Table 2:** Effect of EDU treatment on the growth, total biomass and physiology of wheat cultivars under ambient O_3_ conditions. Here, P_s_: photosynthesis rate, g_s_: stomatal conductance, and Fv/Fm: photosynthetic efficiency. Values are mean ± SE; n = 9. Symbols above the bars represent significant variability between EDU- and non-EDU-treated plants. The one, two and three asterisks indicate the significance levels at * *p* ≤ 0.05, ** *p* ≤ 0.01, and *** *p* ≤ 0.001, and ns is non-significant. Significant variations between cultivars under EDU and non-EDU treatment are respectively represented by different capital and small letters (*p* < 0.05).

Cultivars	Parameters	Non-EDU Treated	EDU-Treated	Significance
**HD 2987**	Plant height (cm)	84.76 ± 0.38 ^b^	97.00 ± 0.57 ^B^	***
	No. of tillers plant^−1^	4.33 ± 0.33 ^b^	6.66 ± 0.33 ^B^	**
	Total biomass (g)	5.96 ± 0.08 ^bc^	7.30 ± 0.15 ^BC^	**
	P_s_ (μmol CO_2_ m^−2^ s^−1^)	16.24 ± 0.83 ^b^	21.84 ± 0.15 ^A^	**
	g_s_ (mol m^−2^ s^−1^)	0.69 ± 0.04 ^a^	0.81 ± 0.01 ^A^	*
	Fv/Fm	0.71 ± 0.01 ^b^	0.79 ± 0.01 ^A^	*
**PBW 502**	Plant height (cm)	85.76 ± 0.58 ^b^	95.20 ± 0.96 ^B^	**
	No. of tillers plant^−1^	4.66 ± 0.33 ^b^	7.33 ± 0.67 ^B^	*
	Total biomass (g)	7.66 ± 0.06 ^ac^	10.30 ± 0.05 ^AC^	***
	P_s_ (μmol CO_2_ m^−2^ s^−1^)	16.48 ± 0.46 ^b^	19.79 ± 1.75 ^A^	ns
	g_s_ (mol m^−2^ s^−1^)	0.50 ± 0.04 ^a^	0.60 ± 0.04 ^B^	*
	Fv/Fm	0.77 ± 0.01 ^ab^	0.82 ± 0.01 ^A^	*
**Kharchiya 65**	Plant height (cm)	125.23 ± 0.66 ^a^	132.96 ± 0.79 ^A^	**
	No. of tillers plant^−1^	7.33 ± 0.67 ^a^	9.67 ± 0.33 ^A^	*
	Total biomass (g)	10.33 ± 0.06 ^ab^	11.60 ± 0.20 ^AB^	**
	P_s_ (μmol CO_2_ m^−2^ s^−1^)	21.07 ± 1.36 ^a^	23.12 ± 1.83 ^A^	ns
	g_s_ (mol m^−2^ s^−1^)	0.62 ± 0.02 ^a^	0.78 ± 0.01 ^A^	**
	Fv/Fm	0.80 ± 0.02 ^a^	0.81 ± 0.00 ^A^	ns

Significant changes (*p* < 0.001) in the total chlorophyll and carotenoids content were prominent in all the EDU-treated cultivars compared to the plants which remained untreated with EDU (Figure 1). Total chlorophyll content maximally increased upon EDU treatment in HD 2987 (77.7%), by 11.1% in PBW 502 and by 36.3% in Kharchiya 65 at 60 days after germination (DAG) (Figure 1). Carotenoids also showed a varied response in all the three test cultivars after EDU treatment. At 60 DAG, the increment was highest in Kharchiya 65 (35.8%), while the increment was lowest in HD 2987 (12.5%) and a reduction of 8.3% was exhibited by PBW 502 (Figure 1). The number of leaves plant^−1^were significantly more in EDU-treated plants compared to non-EDU-treated plants (31, 35 and 18% in HD 2987, PBW 502 and Kharchiya 65, respectively), while the number for senesced leaves plant^−1^decreased at 60 DAG displaying that EDU enhanced the leaf greenness. EDU-treated cultivars showed an obvious decrease in the number of senesced leaves by 75% in HD 2987, 19% in PBW 502 and 47.3% in Kharchiya 65 (Figure 1). Results of the split-plot ANOVA also showed a significant effect (*p* < 0.001) of EDU treatment on the number of leaves; however, the effect was non-significant (*p* = 0.078) for the number for senesced leaves plant^−1^ (Table 1).
